# The first *Conus* genome assembly reveals a primary genetic central dogma of conopeptides in *C. betulinus*

**DOI:** 10.1038/s41421-021-00244-7

**Published:** 2021-02-23

**Authors:** Chao Peng, Yu Huang, Chao Bian, Jia Li, Jie Liu, Kai Zhang, Xinxin You, Zhilong Lin, Yanbin He, Jieming Chen, Yunyun Lv, Zhiqiang Ruan, Xinhui Zhang, Yunhai Yi, Yanping Li, Xueqiang Lin, Ruobo Gu, Junmin Xu, Jia’an Yang, Chongxu Fan, Ge Yao, Ji-Sheng Chen, Hui Jiang, Bingmiao Gao, Qiong Shi

**Affiliations:** 1 Shenzhen Key Lab of Marine Genomics, Guangdong Provincial Key Lab of Molecular Breeding in Marine Economic Animals, BGI Academy of Marine Sciences, BGI Marine, BGI, Shenzhen, Guangdong 518083 China; 2 Key Laboratory of Tropical Translational Medicine of Ministry of Education, Hainan Provincial Key Laboratory of Research and Development of Herbs, School of Pharmacy, Hainan Medical University, Haikou, Hainan 570102 China; 3 BGI Education Center, University of Chinese Academy of Sciences, Shenzhen, Guangdong 518083 China; 4 Center of Reproduction, Development and Aging, Faculty of Health Sciences, University of Macau, Macau, 999078 China; 5 BGI-Shenzhen, BGI, Shenzhen, Guangdong 518083 China; 6 China National GeneBank, BGI, Shenzhen, Guangdong 518120 China; 7 Micro Pharmtech Ltd., Wuhan, Hubei 430000 China; 8 Research Institute of Pharmaceutical Chemistry, Beijing, 102205 China; 9 Laboratory of Fish Genomics, College of Life Sciences and Oceanography, Shenzhen University, Shenzhen, Guangdong 518061 China; 10 Present Address: College of Animal Science and Technology, Zhongkai University of Agriculture and Engineering, Guangzhou, Guangdong 510225 China; 11 Present Address: College of Life Sciences, Neijiang Normal University, Neijiang, Sichuan 641100 China

**Keywords:** Comparative genomics, Proteomic analysis

## Abstract

Although there are various *Conus* species with publicly available transcriptome and proteome data, no genome assembly has been reported yet. Here, using Chinese tubular cone snail (*C. betulinus*) as a representative, we sequenced and assembled the first *Conus* genome with original identification of 133 genome-widely distributed conopeptide genes. After integration of our genomics, transcriptomics, and peptidomics data in the same species, we established a primary genetic central dogma of diverse conopeptides, assuming a rough number ratio of ~1:1:1:10s for the total genes: transcripts: proteins: post-translationally modified peptides. This ratio may be special for this worm-hunting *Conus* species, due to the high diversity of various *Conus* genomes and the big number ranges of conopeptide genes, transcripts, and peptides in previous reports of diverse *Conus* species. Only a fraction (45.9%) of the identified conotopeptide genes from our achieved genome assembly are transcribed with transcriptomic evidence, and few genes individually correspond to multiple transcripts possibly due to intraspecies or mutation-based variances. Variable peptide processing at the proteomic level, generating a big diversity of venom conopeptides with alternative cleavage sites, post-translational modifications, and N-/C-terminal truncations, may explain how the 133 genes and ~123 transcripts can generate thousands of conopeptides in the venom of individual *C. betulinus*. We also predicted many conopeptides with high stereostructural similarities to the putative analgesic ω-MVIIA, addiction therapy AuIB and insecticide ImI, suggesting that our current genome assembly for *C. betulinus* is a valuable genetic resource for high-throughput prediction and development of potential pharmaceuticals.

## Introduction

Cone snails (*Conus* spp.) are a large genus of gastropods that feed on a variety of prey, including worms, snails, and fishes^1,2^. Given that there are ~700 *Conus* species around the world and each possesses over 100 various conopeptides (collectively known as small, bioactive, and heavily post-translationally modified peptides in the *Conus* venom)^1–4^, it has been estimated that there are over 80,000 natural conopeptides^1,5^, of which some have been approved as valuable pharmacological probes and clinical drugs such as the well-known ω-MVIIA (Ziconotide) for treatment of chronic pain in cancer patients^6^. Usually, conopepetides are synthesized in the venom gland as precursor proteins from a single gene that is often comprised of a highly conserved signal peptide, a pro-peptide region, and a hypervariable mature peptide sequence^7^; they have been classified into various superfamilies based on the sequence similarities of their signal peptides.

To date, hundreds of conopeptide genes or conopeptide coding sequences (CDS) have been identified by PCR amplification or purified from crude venom of cone snails using mass chromatography (MS). Moreover, recent high-throughput pipelines of integrating multi-omics (such as transcriptomics and peptidomics^4,8,9^) have dramatically facilitated the discovery of novel conopeptides. In fact, it has been estimated that much less (< 2%) of the total conopeptide diversity has been sequenced^10^. Recently, employing advanced MS systems, several research teams^9,11,12^ have reported the presence of over 1000 different peptides in a single venom, which is a remarkable increase from the early popular estimates of 50–200 conopetides per species. However, great difficulty in extraction of high-quality genomic DNAs has hindered whole-genome sequencing of cone snails^1^, which should have become a valuable resource for comparative examinations of detailed transcription and translation of conopetide genes in single or different *Conus* species.

**Fig. 1:**
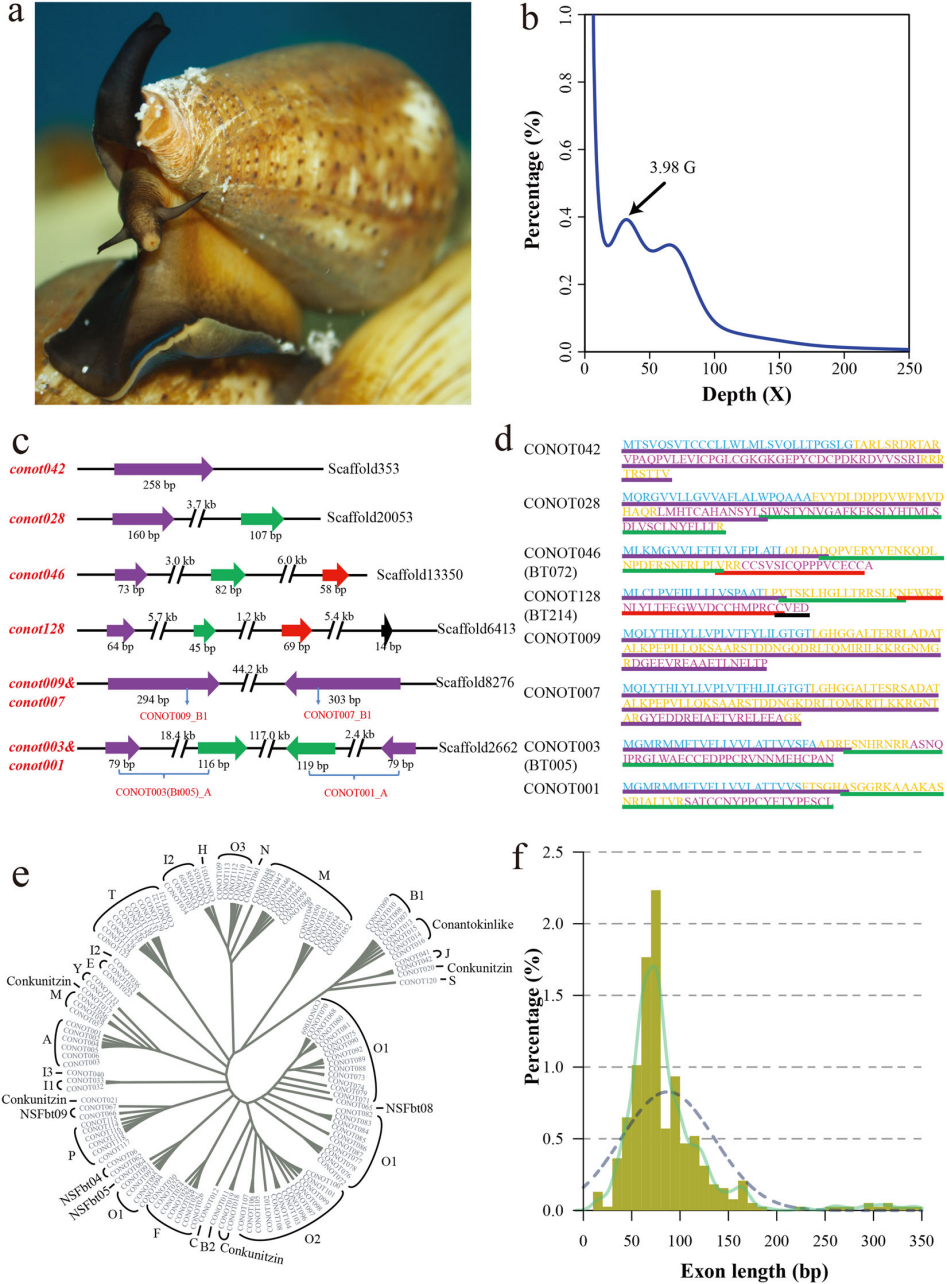
Identification of 133 conopeptide genes from the assembled genome of *C. betulinus*. **a** Image of a representative *C. betulinus*. **b** The 17-mer distribution of sequenced reads. These sequencing data generated from the Illumina short-insert libraries were used for a genome survey analysis^8^. The *x*-axis represents the sequencing depth of each 17-mer, and the *y*-axis represents the percentage of each unique 17-mer. **c** Four representative types of exon distribution in the identified full-length conopeptide genes. Regions in purple, green, red, and black stand for the 1st, 2nd, 3rd, and 4th exons, respectively. **d** Eight deduced protein sequences of these genes in **c**. Sequences highlighted in black, yellow, and red are signal peptides, pro-/post-peptides and mature peptides of these precursors, respectively. **e** Phylogeny of identified conopeptide genes based on predicted signal peptide sequences. **f** Detailed distribution of exon length.

In 2011, a trial next-generation shotgun survey with low quality for *C. bullataus* genome was reported^13^. Recently, targeted sequencing of venom genes from 32 *Conus* genomes^14^ characterized various conopeptide superfamilies. Unpublished genome data for *C. consors* were also deposited at NCBI (under the accession number GCA_004193615.1) for public availability. Here, we generated the first genome assembly for the predominant vermivorous Chinese tubular cone snail (*C. betulinus;* Fig. 1a) in the South China Sea to shed light on the representative genome structure of *Conus* species, and attempted to illustrate a preliminary genetic central dogma of conopeptides for high-throughput development of novel marine drugs. By integration of present genomics and peptidomics data, along with our previously published transcriptomics results in the same species, we tried to establish a rough number ratio for the total genes/transcripts/proteins/post-translationally modified peptides in this representative *Conus* species. Our achieved genome assembly will definitely become a valuable genetic resource for high-throughput prediction of conopeptide genes and transcripts, and lay a solid foundation for in-depth investigations on *Conus* biology and conopeptide pharmacology.

## Results

### Generation of the first *Conus* genome assembly

Specimens of wild middle body-sized^4^ (8.5 ± 0.5 cm in length) *C. betulinus* were collected in the offshore areas of Sanya City, Hainan Province, China. Seven Illumina libraries with various insert-sizes (short: 250, 500, and 800 bp; long: 2, 5, 10, and 20 kb) were constructed^15^ using extracted genomic DNAs from a pool of muscle samples, and then they were sequenced on an Illumina HiSeq 2000 platform (San Diego, CA, USA). After removal of adapter sequences, low-quality raw reads, and PCR duplicates^15^, a total of 188.05-Gb clean reads (59.65% of the 315.24-Gb raw reads) were retained (Supplementary Table S1) for subsequent assembly. Meanwhile, after correction with Illumina short-insert reads, we obtained 225.39 Gb of PacBio corrected long reads (94.26% of the 239.69-Gb raw reads; Supplementary Table S2).

A routine hybrid strategy^16^ was employed to assemble the genome of *C. betulinus*. The final assembly of 3.43 Gb in size, similar to the reported *Conus* estimates by fluorometric assays^14^, accounted for 86.00% of the estimated genome size (3.99 Gb) by a genome survey^15^ in the present study (Fig. 1b; Supplementary Table S3). Its scaffold N50 and contig N50 values are 232.61 kb and 171.48 kb, respectively (Table 1); the genome GC content is 43.80% (Supplementary Fig. S1), and transposable elements (TEs) comprise 38.56% (1.32 Gb) of the achieved genome assembly (Supplementary Table S4). We also evaluated the completeness of our *C. betulinus* assembly with metazoan benchmarking universal single-copy orthologs (BUSCOs)^17^. The high BUSCO value (Supplementary Fig. S2) suggests that our assembled genome covers at least 89.8% of the gene space^15^. Based on the routine annotation strategy^15^ using a combination of transcriptome-based, homologous, and ab initio predictions, we annotated 22,698 protein-coding genes in the genome assembly (Supplementary Table S5).

**Table 1:** Statistics of the genome assembly of *C. betulinus*.

Genome assembly	Parameter
Contig N50 size (bp)	171,480
Contig number (>100 bp)	53,852
Scaffold N50 size (bp)	232,607
Scaffold number (>100 bp)	41,426
The longest scaffold (bp)	2,850,889
Genome GC content	43.80%
Total length (bp)	3,430,828,710
**Genome annotation**	**Parameter**
Protein-coding gene number	22,698
Mean transcript length (bp)	13,448.85
,Mean exons per gene	7.28
Mean exon length (bp)	129.69
Mean intron length (bp)	1,992.04

### Original identification of 133 genome-widely distributed conopeptide genes

After sequence alignment of our previously identified 215 conopeptide transcripts (from a pool of various individuals and tissues; called as Bt001–Bt215)^4^ against the achieved genome assembly, we eventually predicted a total of 133 conopeptide genes (named as *conot001*–*conot133*) with complete sequences of 1–4 exons (Fig. 1c–e; Supplementary Table S6, Fig. S3). The exon sizes range from 14 to 345 bp, with an average length of 87 bp (Fig. 1f).

These predicted conopeptide genes were deduced to corresponding protein sequences, and therefore they are named as “CONOT001–CONOT133” (Supplementary Table S7). Proteins of the same genes and transcripts are termed in the format of “CONOTx(Bty)” (x and y refer to Arabic number suffixes) such as CONOT103(Bt176). These deduced conopeptide proteins can be classified^4,9^ into 21 known superfamilies, 1 cysteine-rich family (Conkunitzin), 1 non-cysteine family (Conantokin-like), and 4 new superfamilies (NSF: NSFbt04, NSFbt05, NSFbt08, and NSFbt09; see Supplementary Table S7).

Fresh muscle tissues were also collected for Hi-C sequencing as previously reported^18^. After obtaining the Hi-C data, we employed Trimmomatic^19^ to remove adapter sequences and low-quality reads. Subsequently, Juicer^20^ was used to align the clean Hi-C reads to the achieved genome assembly for construction of a chromosome-level genome assembly, which is composed of 35 large groups of superscaffolds (Supplementary Figs. S4, S5). Possibly due to the high heterozygosity of *Conus* genomes (see Fig. 1b), this chromosomal assembly is different from a previous report of 16 pairs of chromosomes in *C. magus*^21^. However, genome-wide distribution of conopeptide genes is still obvious after matching these gene sequences onto the assembly, since 46 genes were anchored to 38 positions in 16 different groups (see more details in Supplementary Table S8).

### A primary genetic central dogma of diverse conopeptides

**Fig. 2:**
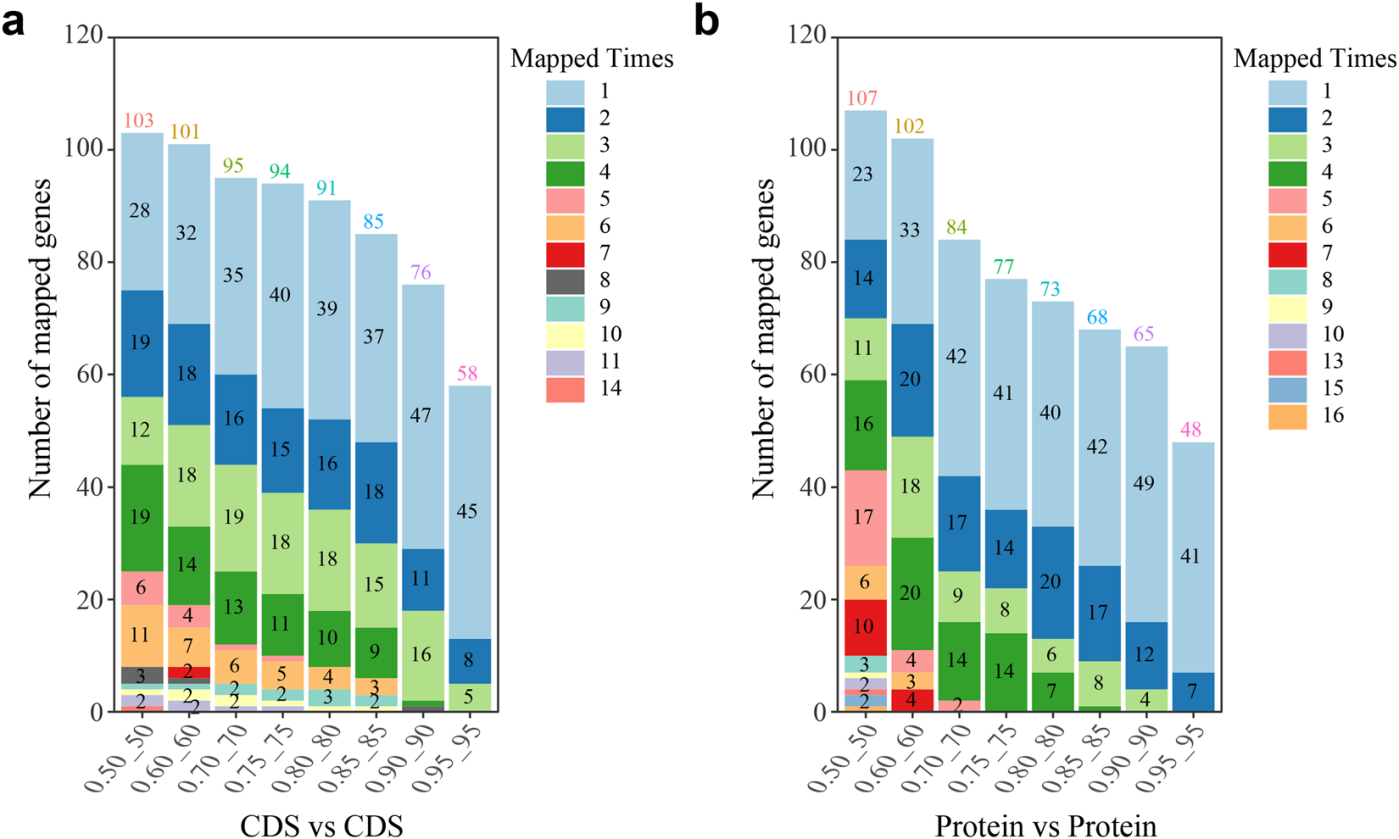
Sequence alignments of conopeptides from one representative *C. betulinus* sample. We used the coding DNA sequences of conopeptide genes (**a**) and relevant protein sequences (**b**) to compare their similarity. The *x*-axis represents the alignment rate and the identity score of each alignment. For instance, 0.50_50 means the alignment rate is 0.50 and the identity score of this alignment is 50. “Mapped Times” represents the mapped number of single CDS/protein against the reference dataset. The number above each stacked bar stands for the total number of CDS/protein that exists in the relevant reference dataset. The number within each bar represents the gene number with “Mapped Times”. Note that the gene numbers at 1 were not denoted in the figures.

The predicted conopeptide genes were aligned to our previously reported 123 conopeptide transcripts from the normalized dataset of a middle body-sized specimen of *C. betulinus*^4^ (Supplementary Table S9). We determined that 127 genes matched to 117 transcripts in the protein sequence alignments (Fig. 2), with parameters set at Query align ratio ≥ 0.80, Subject align ratio ≥ 0.80 and identity ≥ 80 (Supplementary Table S7). When the 133 predicted conopeptide genes were aligned to our previously reported mixture of 215 transcript sequences in *C. betulinus*^4^, a total of 80 potentially corresponding transcripts were determined (Supplementary Table S6); however, few genes (such as *conot009* and *conot026*) may correspond to multiple transcripts individually.

The primary number ratio of entire conopeptide genes to total transcripts is almost 1:1, since a total of 123 transcripts were identified in a similarly middle body-sized snail in our previous report^4^. However, we have also noted that only a fraction (61/133 = 45.9%) of the identified conotopeptide genes from the achieved genome assembly are transcribed with transcriptomic evidence (see more details in Supplementary Table S10). Usually, one transcribed conopeptide gene was predicted with sole transcript; however, certain genes (such as *conot009*) correspond to multiple transcripts, possible due to high intraspecific differences (using different samples for the genome and transcriptome sequencing) or mutation-based variances^14^ (see more detailed explanations under the subsequent section of “Discussion”). When conopeptide superfamilies were considered, we note that the corresponding proportion of transcribed genes was elevated up to 53.1% per superfamily (Supplementary Table S11).

**Fig. 3:**
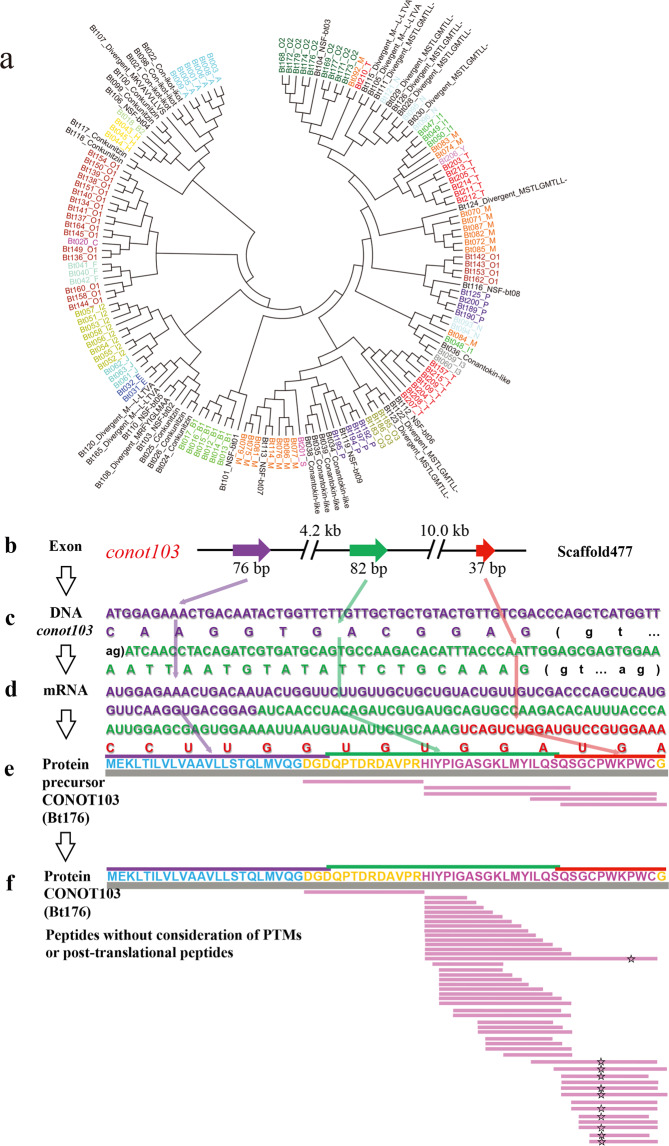
Identified conopeptide transcripts and post-transcriptional peptides. **a** Phylogeny of previously deduced conopeptide precursors with full-length signal peptides^4^. **b**−**e** Central dogma of the representative conopeptide CONOT103(Bt176). Regions in purple, green, and red represent the 1st, 2nd, and 3rd exons, or the corresponding sequences, respectively. Sequences highlighted in blue, orange, and red are signal peptides, pro-/post-peptides, and mature peptides of the precursor, respectively. **f** Functional conopeptides with post-translational modifications and/or N- and C-terminal truncations^7^ that were detected by MS. Amino acid sequences with open star illustrate amino acid modifications at hydroxy-prolines (P).

To investigate the potential relationships among our 215 previously identified conopeptide transcripts from *C. betulinus*^4^, we constructed a phylogenetic tree based on their signal peptide sequences (Fig. 3a). We observed that most of these conopeptides from the same predicted superfamily are clustered together, such as A, B_1_, and O_2_ superfamilies. However, some predicted superfamilies can not be clustered well possibly due to high sequence diversity. For example, the putative M superfamily was divided into 3 groups except for Bt084 and Bt092. Interestingly, NSFbt03, NSFbt07/NSFbt08, and NSFbt09 were embedded in the O_2_, M, and P superfamilies, respectively, indicating their high similarity (although less than 80%) to each corresponding superfamily.

**Fig. 4:**
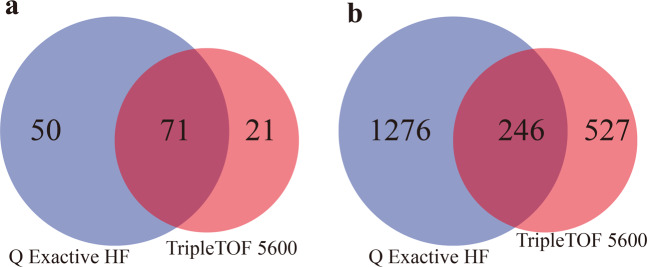
Analysis of peptidomics data of *C. betulinus* venom by two different mass spectrometry (MS) systems. **a** Numbers of identified proteins. **b** Numbers of identified peptides.

Meanwhile, we employed two advanced MS systems (TripleTOF 5600 and Q Exactive HF) to perform peptidomics studies (see more details in “Materials and methods”) for exploration of various conopeptides in the venom gland of *C. betulinus*. Using deduced protein sequences of the 215 previously identified transcripts as references^9^, we discovered that 1522 peptides, matching 121 conopeptide transcripts, were detected by the Q Exactive HF (Supplementary Table S12) when post-translational modifications (PTMs) were not considered. Similarly, 773 peptides, matching 92 conopeptide transcripts, were identified by the TripleTOF 5600 without consideration of PTMs (Supplementary Table S13). However, only 282 (31.8%) peptides representing 71 (77.2%) transcripts were identified to be overlapped when using both methods (Fig. 4). By the way, the credibility of conopeptide identification is guaranteed. For example, BT112 is such a short peptide (CFCLTR); however, its peptide spectrum match (PTM) map (Supplementary Fig. S6) proves that the identification is indeed reliable.

Overall, at least 142 proteins based on 2474 conopeptides with PTMs or 2049 peptide sequences without consideration of PTMs (Supplementary Tables S14, S15, S16) were identified from the two peptidomics methods, suggesting a number ratio of ~1:10s for transcripts or proteins to venom conopeptides. This primary ratio is somehow consistent with a previous report in *C. marmoreus*^7^, which revealed 2710–6254 peptides using various MS systems (including the high-sensitivity TripleTOF 5600) and matched them to most of the ~100 transcripts. However, we should realize that different conopeptide proteins may correspond to various numbers of venom peptides (Supplementary Tables S14, S15).

### High-throughput predictions for development of potential pharmaceuticals

The well-known conopeptides ω-MVIIA, AuIB and ImI were first isolated from the venom of corresponding cone snails^6,8,22,23^. The most famous calcium channel blocker ω-MVIIA (Ziconotide) was approved by the US Food and Drug Administration in 2004^6^. AuIB, as one of the 12 reported conopeptides with anti-addictive activity (Supplementary Table S17), specifically inhibits neuronal nicotinic acetylcholine receptors (nAChR; α3β4 subtype)^22^. ImI has been reported to block nAChRs in fruit fly (*Drosophila melanogaster*) with an insecticidal activity^8,23^.

**Fig. 5:**
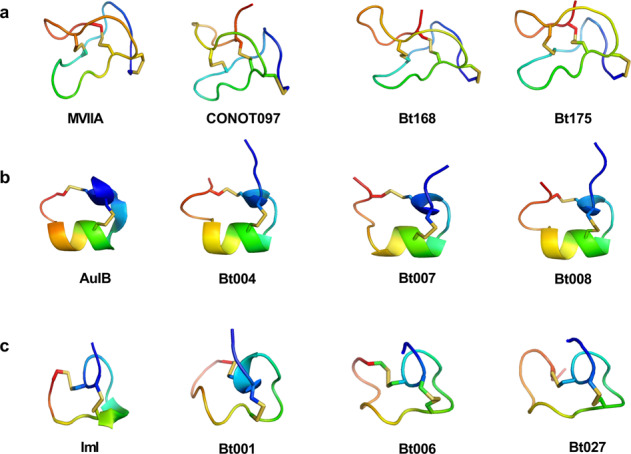
Predicted stereostructures of homologous conopeptides on the basis of MVIIA. Stereostructures of MVIIA (**a**), AuIB (**b**) and ImI (**c**). Each top 3 best-hit conopeptides from homologous alignments were selected to predict stereostructures. Models of the conopeptides were built using MVIIA (PDB:1OMG), AuIB (PDB:1MXN) and ImI (PDB:2BC7) as templates in MODELLER (version 9v12). All the modeling structures were generated with PyMol. See more details about the protein sequence alignments in Table 2.

Based on the reported peptide sequences of MVIIA, AuIB and ImI (Supplementary Table S18), we searched the 133 conopeptide genes and the 215 transcripts^4^ of *C. betulinus* by using the routine homologous sequence alignment^8^. A total of 13 representative conopeptides were obtained, which ranged from 17 to 28 amino acid residues (aa) in length (Supplementary Table S18). A homologous modeling method^8,24^ was used to predict 3D structures of these homologous conopeptides, confirming their similar stereostructures to MVIIA, AuIB, and ImI, respectively (Fig. 5). Our data suggest that these novel conopeptides may be potentially developed as valuable analgesics, addiction therapies, and insecticides, respectively (Table 2; Supplementary Table S18).

**Table 2:** Protein sequence alignments of the representative conopeptides.

Name	Conotoxin sequence	GenBank accession no.
(a) *Analgesic activity*		
MVIIA		P05484.2
CONOT097		--- (Present study)
Bt168		KU563979.1
Bt175		KU564050.1
(b) *Antiaddictive activity*		
AuIB		P56640.2
Bt007		KU564009.1
Bt004		KU564008.1
Bt008		KU317629.1
(c) *Insecticidal activity*		
ImI		KJ801971.1
Bt001		KU563886.1
Bt006		KU563888.1
Bt027		KU564013.1

The list is simplified from Supplementary Table S18 for a 3D-structural comparison in Fig. 5.

Note: Conserved residues (with similar properties) among different conopeptides are highlighted in the same background color. *, Amidated C-terminus.

For the homologous sequences of MVIIA (Supplementary Table S18a), these all contain the representative backbone of six cysteines (C) and one glycine (G) (C-G-C-CC-C-C; Table 2a and Fig. 5a). The six cysteine residues are linked by 3 disulphide bonds, serving to stabilize the conformation and to form 4 loops. Similarly, the homologous sequences of AuIB (Supplementary Table S18b) have the representative backbone of 4 cysteines and 2 prolines (P) (CC-P-C-P-C; Table 2b and Fig. 5b). The homologous sequences of ImI in Supplementary Table S18c have the representative backbone of CC-P-C-C (Table 2c and Fig. 5c). Four cysteine residues are linked by 2 pairs of disulphide bonds, in which loop 1 (between the 1st and the 3rd cysteines) and loop 2 (between the 2nd and the 4th cysteines) are variable to determine the selectivity of corresponding nAChR subtypes^1,8^.

## Discussion

Next-generation high-throughput sequencing technologies have opened up new opportunities for genome-wide studies on various animals, plants, and bacteria. In the present study, we combined Illumina short reads and PacBio long reads to generate the first *Conus* genome assembly, which revealed a primary genetic central dogma of conopeptides in the representative *C. betulinus*.

In fact, an early attempt to sequence *C. bullataus* genome by Olivera and colleagues^13^ was published in 2011. Although this is a trial shotgun survey with low quality, the authors provided many detailed characterizations. For example, they randomly selected 30 million genomic reads and determined the GC content to be 42.88%, which is similar to our genome-wide calculation of 43.80% for the *C. betulinus* genome (Supplementary Fig. S1). Using genomic and transcriptomic reads, the authors had developed a novel method to estimate genome size^13^; the final estimate for the size of *C. bullataus* genome was 2.56 Gb, which is less than our assembly (3.43 Gb) and estimate (3.99 Gb) for the *C. betulinus* genome. This difference also supports the high diversity of various *Conus* genomes.

Recently, Phuong and Mahardika^14^ successfully recovered venom gene superfamilies from the genomes of 32 cone snails by using targeted sequencing techniques. They found that conopeptide gene superfamilies are composed of 1–6 exons (covering our present report of 1–4 exons for *C. betulinus*; see more representative details in Fig. 1c), and single exon is typically short in length (5–444 bp; covering our current range of 14–355 bp for *C. betulinus*) with an average length of 85 bp (similar to the average of 87 bp for *C. betulinus*; see Fig. 1f). Interestingly, the detailed exon length distribution from the big dataset of 32 *Conus* species is almost the same as our data in Fig. 1f, providing a solid support to our present high-quality genome assembly for *C. betulinus*. This recent work also improved our understanding of conopeptide molecular evolution. Based on the extracted genomic data, the authors identified variable conotoxin gene copies from 120 (*C. papilliferus*) to 859 (*C. coronatus*), which cover our present prediction of 133 conotoxin genes in the *C. betulinus* genome. Although they stated that diet specificity did not predict patterns of conopeptide evolution, we observed a primary evolutionary pattern for those species within the same phylogenetic clade. It somehow seems to be true that a primitive *Conus* species usually owns less conopeptide gene copies (see more details in the Fig. 5 of Phuong and Mahardika^14^), which is consistent with the low gene copy number (133) and the proposed primitive status^2^ for the vermivorous *C. betulinus*.

As reported previously^14,25^, only a fraction of conopeptide genes in *Conus* genomes were proved to be transcribed when compared transcriptome and genome sequences. In our present study, the entire gene number (133) is slightly higher than the sum of identified transcripts (~123) in individual cone snail. By comparing the transcriptome data to the 133 predicted conopeptide genes, we figured out how many genes transcribed in the mixed and individual samples, respectively. Our results showed that 87 (65.4%) conopeptide genes are transcribed in the pooled samples (Supplementary Table S6). The proportion of conopeptide genes transcribed per gene superfamily was up to 72.5% (Supplementary Table S11). However, in the individual middle-sized cone snail, only 61 (45.9%) conopeptide genes are transcribed with transcriptomic evidence (Supplementary Table S10). Such transcription pattern was also comparable to the previously reported proportions of 24%–63% from 32 various *Conus* species^14^, confirming that only a fraction of conopeptide genes are transcribed. This discrepancy in genome and transcriptome data for conopeptides may be caused by variable parameters in life stage and geographic localization, or by the genome itself since many genes are no longer functional (i.e., pseudogenized), or by different specimens or mutation-based variances^14^. We may be able to improve the present version of genome assembly with more PacBio and Hi-C sequencing reads; in this case, few more conopepetide genes are expected to be identified, which can increase the align number to corresponding transcripts.

Dramatic interspecies and even intraspecies variations have been reported previously^9,11,26–28^. Milked venom has been thought to be perfect for subsequent MS examination, since it lacks cellular debris and unprocessed toxins^9,26^; however, production of different venoms by same individual was also revealed as an unsuspected contribution to venom diversity^26^. In the present study, however, we had to apply the routine and conveniently dissected venoms, which may have complicated the component profile of venom conopeptides. High diversity in conopeptide transcripts were also extensively documented in various *Conus* species^9,27,28^, with a big range from 100 up to 522 (or more)^9^ that covers our individual 123 transcripts for *C. betulinus*. These accumulated data indicate that the number ratios of total conopetpide genes: transcripts: peptides in various cone snails may present somewhat variances. Therefore, our present ratio for *C. betulinus* may be temporarily special. More studies and samples are required to confirm these primary data, although they may be relatively stable in this species.

Recent estimations of venom conopeptide number at the proteomic level generate an increasing value to several thousands^9^ using highly sensitive MS systems. Such discrepancy between transcriptomic and proteomic data in terms of sequence could be explained not only by post-translational process^9^, but also by degenerated products^27^ (although difficult for determination). As reported in detail by Dutertre et al.^9^, variable peptide processing at the proteomic level, generating a big diversity of conopeptides with alternative cleavage sites, post-translational modifications, and N-/C-terminal truncations, may explain how a limited set of ~100 transcripts can generate thousands of conopeptides in the venom of individual cone snail.

**Fig. 6:**
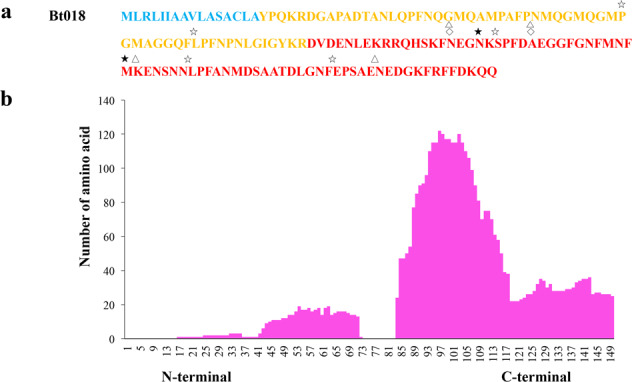
Protein sequence and mapping graph of the representative conopeptide Bt018. **a** Protein sequence of the Bt018. Blue marks the signal peptide region, and amino acid sequences with bold font depict those peptides that were identified by MS. Amino acid sequences with open star, filled star, open triangle, open diamond on the top illustrate amino acid Hydroxy (P), Hydroxy (K), Gamma-carboxy-glutamic (E), and Pyroglutamic (E) modifications (identified by MS), respectively. **b** The mapping graph was generated by the PepEx. Height of each column represents the number of amino acids for the 225 corresponding peptides.

In the present study, additional peptide diversity was often created by PTMs^9^ (Supplementary Tables S13, S14; not easily be predicted from precursor sequences), such as at 4-hydroxy-proline (P), 5-hydroxy-lysine (K), gamma-carboxyglutamic acid (E), and pyroglutamic acid (E). According to the total modifications of these detected peptides (Supplementary Table S14), we estimate that the maximal number of conopeptides may reach 6653. Interestingly, conopeptide Bt018 may have the maximal (up to 4608) modified combinations because it matches to a higher number of peptides (225) and involves more modifications (4 different modifications at 11 amino acid sites, and some sites with different types of modification; Supplementary Table S16). Related protein coverage areas, number of identified peptides, and modification sites of the representative conopeptide Bt018 are summarized in Fig. 6. In summary, taking the number of conopeptide genes identified in the genome assembly into consideration, we assume that the number ratio of genes: transcripts: precursors: post-translationally modified peptides of conopeptides is around 1:1:1:10s. As stated in a previous report^9^, the vast majority of detected conopeptides are generated from a more limited set of genes/precursors through peptide processing, which produces various conopeptides with alternative cleavage sites, post-translational modifications, and highly variable N-/C-terminal truncations (see the representative Fig. 3f). This variable peptide processing, along with potentially intraspecies variation^4,9^, may explain how 133 genes (Supplementary Table S6) and ~123 transcripts (Supplementary Table S7) can generate thousands of conopeptides in the venom of individual cone snail.

**Fig. 7:**
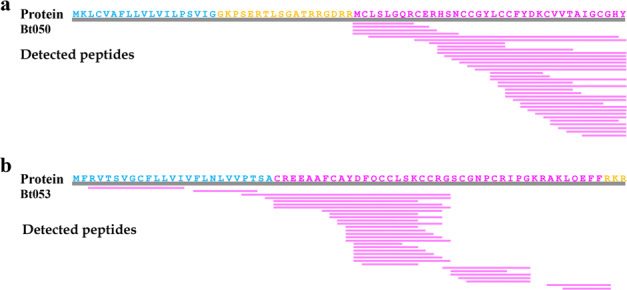
Mapping examples of sequenced peptides to the examined conopeptides. Representative peptides are Bt050 (**a**) and Bt053 (**b**). Sequences highlighted in blue, orange, and rosy red are signal peptides, pro-/post-peptides, and mature peptides, respectively.

Recovery of intact conopeptide proteins from the detected peptides prompted us to look back into the assembled genome and to search for a complete central dogma of conopeptides. Here, we illuminated 9 representative conopeptides, such as CONOT006(Bt003), CONOT019(Bt025), CONOT039(Bt057), CONOT046 (Bt072), CONOT52(Bt075), CONOT055(Bt077), CONOT071(Bt150), CONOT103 (Bt176) and CONOT129(Bt207), with both full-length exons and complete mature peptides as examples. In fact, Bt176 was encoded by the *conot103* gene, which is localized on the Scaffold477 with 3 exons (Fig. 3b). The transcribed mRNA is 195 bp in length, with a stop codon (UGA) at the end. The full length of deduced precursor consists of 64 aa, in which 1−26th, 26−53rd, and 53−64th aa are encoded by the exons 1, 2, and 3, respectively (Fig. 3b–d). Meanwhile, the pro-peptide (24−36th aa), mature peptide (37−63rd aa), and post-peptide (64th aa) have also been detected by the 2 peptidomics methods (Fig. 3e). However, some conopeptides can only be detected in the mature peptide regions (Fig. 7a; such as Bt050), whereas some signal peptide regions can also be detected by the peptidomics sequencing (Fig. 7b; such as Bt053). Meanwhile, Bt018 and CONOT103(Bt176) were detected with a total of 225 and 46 post-translationally modified peptides, respectively; their high transcription levels, revealed in our previous report^4^ (Supplementary Table S19), concurrently support their critical importance in prey capture and/or defense^29,30^.

**Fig. 8:**
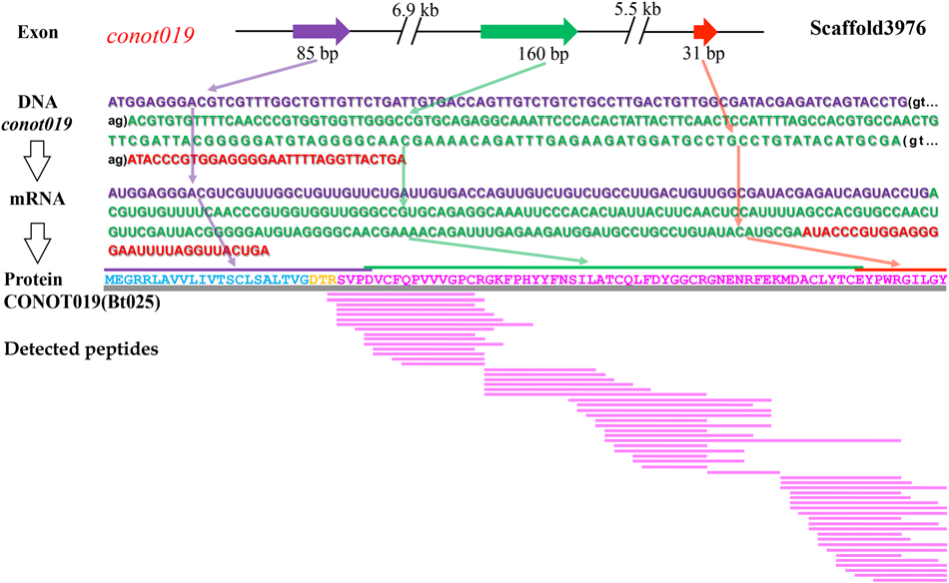
A representative central dogma for the conopeptide Bt025. Regions in purple, green, and red colors represent the first, second and third exons, or their corresponding DNA/mRNA sequences, respectively. Sequences highlighted in blue, orange, and rosy red are signal peptides, pro-peptides, and mature peptides, respectively.

As discussed in previous reports^9^, the high sensitivity of the TripleTOF system allowed fine characterization of many peptide variants for each conopeptide transcript. However, most of the peptide diversity corresponded to truncated forms of the mature peptide or pro-peptide sequences. It is interesting to observe that conopeptide proteins tend to be digested at several favorite cleavage sites (Fig. 8). We used CONOT019(Bt025) as an example to examine which enzymes and what kind of cleavage sites^31^ are preferred in a precursor. Our results proved that there are 35 possible cleavage sites in this protein sequence, which could be digested by 18 different enzymes (Supplementary Table S20). The enzyme with the most cleavage sites (*n* = 15) is proteinase K, which preferentially cleaves aa residues^18^ of A, E, F, I, L, T, V, W or Y. However, the 41^st^ R of the representative Bt025 is the most cleaved site (with 12 detected peptides; see Supplementary Table S20) that could be digested by 3 various enzymes, including Arg-C proteinase, clostripain and trypsin.

In summary, this is the first report of a *Conus* genome assembly. With the integration of multi-omics (genomics, transcriptomics and peptidomics) data, we attempted to illustrate the special genetic central dogma of conopeptides in *C. betulinus*, assuming that the primary number ratio for total conopeptide genes: transcripts: proteins: post-translationally modified peptides is ~1:1:1:10s. Some predicted conopeptides in our present study are similar to the well-known analgesic ω-MVIIA, addiction therapy AuIB and insecticide ImI in cysteine backbone and 3D structure, suggesting that these novel peptides could be developed as pharmaceutical candidates.

## Materials and methods

### Genome sequencing and assembly

Specimens of wild *C. betulinus*, 8–9 cm in length, were collected from offshore areas of Sanya City, Hainan Province, China. We employed two different strategies to sequence the genome of this popular *Conus* species in the South China Sea.

#### Illumina sequencing

Genomic DNA was extracted from the pooled muscle of two specimens using Qiagen GenomicTip100 (Qiagen, Gaithersburg, MD, USA). In accordance with the standard protocol from Illumina (San Diego, CA, USA), we constructed three short-insert libraries (250, 500, and 800 bp) and four long-insert libraries (2, 5, 10, and 20 kb) for sequencing on an Illumina HiSeq 2000 system^15^. In total, we generated 315.24 Gb of raw reads (Supplementary Table S1). SOAPfilter v2.2^32^ with default parameters was employed to collect clean data by removal of low-quality reads (including reads with 10 or more nonsequenced/low-quality bases), PCR duplicates and adapter sequences.

#### PacBio sequencing

Genomic DNA was extracted from the muscle of another specimen for construction of a 20-kb PacBio SMRT libraries, which were sequenced by a PacBio Bioscience Sequel platform (Menlo Park, CA, USA). Finally, we obtained a total of 239.69 Gb of long reads (Supplementary Table S2).

#### Genome assembly

We estimated the genome size to be 3.98 Gb (Fig. 1b; Supplementary Table S3) on the basis of the 17-mer depth frequency distribution method^15^. A routine hybrid strategy^16^ was applied to assemble the genome sequences. First, SparseAssembler^33^ with optimized parameters (K 51 NodeCovTh 2 EdgeCovTh 1) was performed to generate a *De Bruijin* graph based original contigs by using the Illumina short-insert reads. Subsequently, we employed the DBG2OLC^34^ program to remap these contigs upon the PacBio corrected long reads for construction of longer contigs. We then used Minimap v2^35^ and Racon v1.3.1^36^ to generate consensus contigs. Finally, we employed SSPACE_Standard v3.0^37^ and SSPACE-LongRead v1-1^38^ to obtain a more continuous assembly.

#### Genome annotation

Homology-based predictions were performed to detect known transposable elements (TEs) by running RepeatMasker (version 3.3.0)^39^ against the RepBase TE library (release 17.01)^40^. Repeat sequences at DNA and protein levels were identified using the TE library and RepeatProteinMask^39^. We constructed de novo repeat library using LTR_FINDER^41^ and RepeatModeler^39^ (version 1.0.5), and then used RepeatMasker^39^ to obtain TE results with detailed classifications for each repeat family.

Tandem repeats were also searched in the archieved genome assembly using Tandem Repeats Finder (TRF, version 4.04)^42^. All the repeat sequences were finally combined with non-redundant repetitive sequences (Supplementary Table S4). Protein coding genes were predicted by combination of de novo, homology, and transcriptome^4^ based methods^15^. All the predicted genes (Supplementary Table S5) were integrated into a comprehensive and non-redundant gene set using Maker-2.31.8^43^.

#### Genome assembly evaluation

We evaluated the completeness^15^ of our *C. betulinus* assembly with metazoan and eukaryotic benchmarking universal single-copy orthologs (BUSCOs v3.0^17^).

### Screening of conopeptide genes from the assembled genome

#### Identification of conopeptide genes

A total of 215 conopeptide transcripts were previously identified by us from 6 transcriptome datasets of *C. betulinus*^4^. Corresponding cDNA sequences were mapped against our present genome assembly using Blast^44^ and Genewise^45^. Subsequently, the alignment sequences of scaffolds were extracted using a Perl script based on high similarity.

With reference to the corresponding transcripts, these sequences were divided into exon and intron regions (Supplementary Table S6) to construct conopeptide/conotoxin genes (named as *conot001*–*conot133*) manually. These predicted genes were then translated into corresponding proteins (precursors) and named as “CONOT001–CONOT133” (Supplementary Table S7). Proteins of the same genes and transcripts are termed in the format of ‘CONOTx + (Bty)’ (x and y refer to Arabic number suffixes), such as CONOT103(Bt176).

Based on at least 75% identity in the conserved signal peptide sequences^10,46^, these translated protein sequences were assigned to various conopeptide superfamilies and groups^4^.

#### Phylogenetic analysis of the identified conopeptides

Complete signal peptide sequences of the 133 genes and 215 transcripts were manually selected. They were then aligned online using Multalin^47^ (v 5.4.1). Phylogenetic trees were constructed by using MEGA6^48^ with the maximum likelihood method. Statistical supports were assessed with 1000 bootstrap pseudo-replicates, and those classic superfamilies were marked with different colors (Fig. 1e).

#### Gene and transcript analysis

The predicted 133 genes (*conot001*–*conot133*) were realigned onto the 215 transcripts^4^ using BLAST with an E-value < 1.0e^−5^ and an alignment rate >0.6. Solar v0.9.6^49^ was conducted to link high-identity segment pairs. Those low-quality sequences with alignment rate < 0.5 and mapping identity < 0.5 were discarded.

To characterize the detailed proportion of transcribed conopeptide genes, we mapped nucleotide sequences of the totally identified 215 transcripts^4^ to the CDS of 133 conopeptide genes by restricting the blast alignment ratio ≥ 95% and the sequence identity ≥ 95%. These settings of such stringent cutoffs were due to the high conservation of signal peptides and the good similarity of conopeptide genes within a superfamily^14^. We also calculated the number of transcribed conopeptide genes in the middle body-sized snail (with 123 identified transcripts), which potentially represents the transcription pattern in single snail.

### Hi-C sequencing and assembly

#### Hi-C library construction

Fresh muscle tissues were collected for the Hi-C sequencing as described in the previous studies^18^. In brief, ~1 g of muscle sample was cross-linked with 1% formaldehyde for 10 min at room temperature and quenched with glycine (final concentration of 0.2 M) for 5 min. The cross-linked cells were subsequently lysed to extract nuclei. The DNAs in nuclei were further digested with *Mbo*I and marked with biotin-14-dCTP (Invitrogen, Carlsbad, CA, USA) and then ligated by T4 DNA ligase. After reversing cross-links, the ligated DNA was sheared to 300- to 500-bp fragments. The DNA fragments were purified through biotin-streptavidin mediated pull-down and were further blunt-end repaired and A-tailed using NEBNext Ultra II DNA library Prep Kit (New England Biolabs, Ipswitch, MA, USA) according to manufacturers’ instructions. Finally, the Hi-C libraries were quantified and sequenced on the Illumina HiSeq X Ten platform (San Diego, CA, USA) with the 150 paired-end mode.

#### Hi-C Proximity-guided assembly

After obtaining Hi-C reads, we employed Trimmomatic (version 0.38)^19^ to remove the adapter sequences and low-quality reads. Then, Juicer v1.6.2^20^ was used to align Hi-C reads to the achieved genome to obtain the connection relationship between contigs, and 3d-dna (version180922)^50^ was used to scaffold those contigs through the misjoin correction algorithm, the scaffolding algorithm, and the merging algorithm using default parameters. The chromosome-level genome assembly was achieved after polishing, sealing, and chromosome splitting of mega-scaffolds. Finally, misassembled contigs in visual were corrected using JucieBox (v 1.8.8)^51^. Align the identified 133 gene sequences to the 35 large groups using blastn (version 2.7.1+) with the following parameters: blastn -query gene.fasta -db all_group.fasta -outfmt 5 -out xx.out -evalue 1e^−5^.

### Peptidomics sequencing to identify venom conopeptides

#### Venom collection

Venom ducts of collected *C. betulinus* (*n* = 3) were dissected quickly on ice for subsequent homogenization^52^ in 30% acetonitrile (ACN) containing 0.1% trifluoroacetic acid (TFA)^53,54^. Protease inhibitor cocktail (Roche, Shanghai, China) was added to prevent degradation of proteins and peptides according to the manufacturer’s instructions^55^. After centrifugation of 16,100× *g* at 4 °C for 30 min, the supernatant (crude venom) was collected for lyophilization and final storage at −20 °C until use.

#### Preparation of venom samples

Crude venom was suspended in 0.1 M Tris-HCl (pH 8.5) with 8 M urea. Protein concentrations were measured using the standard Bradford method. Denatured proteins and peptides were reduced with 10 mM dithiothreitol (DTT) at 56°C for 45 min. After cooling to room temperature, the solutions were alkylated with 55 mM Iodoacetamide (IAA) in dark at room temperature for 30 min. Venom peptides were extracted and purified using the Strata-X C18 column (Phenomenex Inc., Torrance, CA, USA) according to the manufacturer’s instructions. The final eluates (venom peptides) were dried in a ScanVac freeze drier (LaboGene, Lynge, Denmark) for storage at −20 °C before use. Venom peptides were quantified using a NanoDrop A280 system^56^ (Thermo Fisher Scientific, Waltham, MA, USA).

#### Peptide fractionation

Two different HPLC approaches were applied, including strong cation exchange (SCX) and high-pH reverse phase (Hp-RP) chromatography. In the Hp-RP procedure, 100 μg of venom peptides was separated by a Gemini Hp-RP column (4.6 × 250 mm, 5 μm, 110 Å; Phenomenex Inc.). Fractionation was performed using a linear gradient of 0%–40% of buffer A (80% ACN, 20 mM NH_4_FA, pH 10) at a flow rate of 1 mL/min for 40 min, 40%–90% of buffer A for 2 min, and 90% of buffer A for 3 min. In the SCX HPLC procedure, 100 μg of venom peptides was separated by a Luna SCX column (4.6 × 250 mm, 5 μm, 110 Å; Phenomenex Inc.). Fractionation was performed using a linear gradient of 0%–40% of buffer B (25% ACN, 1 M KCl, 10 mM KH_2_PO_4_, pH 3.0) at a flow rate of 1 mL/min for 40 min, 40%–90% of buffer B for 2 min, and 90% of buffer B for 3 min. All the HPLC procedures were manipulated in a 20AD HPLC system (Shimadzu, Kyoto, Japan). Absorbance was monitored at 214 nm, and the fractions were collected along the gradient for lyophilization.

#### Liquid chromatography-tandem MS (LC-MS/MS) Analysis

##### TripleTOF 5600

LC-MS/MS was performed using a TripleTOF 5600 MS System (AB Sciex, Foster City, CA, USA) coupled to a nano-HPLC system (Shimadzu). The peptides of each fraction from the SCX were separated by nano-HPLC on an in-house packed 12 cm × 75 μm Ultimate XB-C18 column (3 μm, 120 Å; Welch Materials Inc., Shanghai, China) at a flow rate of 300 nL/min. Each fraction was loaded in buffer C (5% ACN, 0.1% formic acid (FA)) and eluted with a linear 40-min gradient of 5%–45% buffer D (95% ACN, 0.1% FA). MS parameters were set as follows: electrospray voltage of 2.5 kV, positive ion data-dependent scan mode, full scan range of 350–1500 *m/z*, selection of the top 30 ions, and dynamic exclusion duration 18 s.

##### Q Exactive HF

Another LC-MS/MS was performed using a Q Exactive HF coupled to an UltiMate 3000 UHPLC system (Thermo Scientific). The peptides of each fraction from the Hp-RP were separated by a 75 μm × 25 cm in-house analytical column that packed with Ultimate LP-C18 particles (3 μm, 120 Å; Materials Inc.) at a flow rate of 300 nL/min. Each fraction was loaded on a trap column (30 μm × 5 mm, μ-Precolumn; Thermo Fisher Scientific) with buffer E (2% ACN, 0.1% FA) in 5 min, followed by a linear 40-min gradient of 5%–35% buffer F (98% ACN, 0.1% FA), and then increased to 80% in 5 min. Mass spectrometry data were acquired with a top30 data-dependent mode scan method. The electrospray voltage was set to1.6 kV and full scan range was set to 350–1600 *m/z*. We used a resolution of 60,000 at *m/z* 200 for survey scans. Precursor ions were fragmented by high-energy collisional dissociation (NCE 27%), and fragment ions were detected in the Orbitrap (R = 15,000 at *m/z* 200). Dynamic exclusion duration was set to 30 s.

#### Peptide Identification

The MS/MS data were converted into a Mascot Generic Format (MGF) file using Proteome Discoverer 1.4 (Thermo Fisher Scientific) for the Q Exactive HF and using MS Data Converter v1.3 (AB Sciex, Foster City, CA, USA) for the TripleTOF 5600. The MGF data were searched by Mascot (version 2.3.02; MatrixScience, Boston, MA, USA) against our previous transcriptome database^4^. Enzyme was set to none, with Carbamidomethyl (C) as a fixed modification, and Oxidation (M), Gln->pyro-Glu (N-term Q), Deamidated (NQ), 4-hydroxy-proline (P), 5-hydroxy-lysine (K), bromotryptophan (W), gamma-carboxy-glutamic acid (E), pyroglutamic acid (E), sulfotyrosine (Y), and gamma-hydroxy-D-valine (V) as variable modifications^34^. Decoy database was selected. Peptide mass tolerance was set to 10 ppm and MS/MS tolerance was set to 0.02 Da for the Q Exactive HF; peptide mass tolerance was set to 0.05 Da and MS/MS tolerance was set to 0.1 Da for the TripleTOF 5600.

Finally, the peptides were filtered at e-value < 0.05 for the false discovery rate (FDR) using the Mascot searching engine; that is to say, the ion scores were higher than corresponding identity scores^57,58^. The manual method and the PepEx (https://github.com/ eparker05/PeptideExtractor) were both used to map the distribution of peptides onto their full-length conopeptide protein sequences. Protein modification sites and types were summarized by a perl script, and all possible combinations of the modified peptides are proposed on the basis of these determined modification parameters. PSM images were visualized by PDV^59^.

### Bioactivity prediction for determined conopeptides

Based on the reported MVIIA, AuIB and ImI sequences^23,24,60^, homologous alignment was performed to screen peptides with potentially related activities from the determined 133 genes and 215 transcripts^4^. These sequences were aligned for comparison by using MEGA6 and GeneDoc 2.7 (https://github.com/karlnicholas/GeneDoc). To represent identical or homologous residues in each sequence, amino acids were marked with different colors.

The 3D structures of MVIIA, AuIB and ImI were downloaded from the public PDB database (http://www.wwpdb.org/). Models of the conopeptides were built using the MVIIA (PDB:1OMG), AuIB (PDB:1MXN) and ImI (PDB:2BC7) as templates in MODELLER (version 9v12)^61,62^ as described previously^8^. All modeling images were generated with PyMol (http://www.pymol.org).

## Supplementary information

Supplementary information

Table S6

Table S7

Table S9

Table S10

Table S11

Table S12

Table S13

Table S14

Table S15

Table S16

## Data Availability

Supporting datasets are included within this article and its Supplementary Information. The genome reads generated in this study have been deposited in China National GeneBank Nucleotide Sequence Archive (NSA) with accession IDs from CNX0040469 to CNX0040494 and from CNS0048937 to CNS0048939 under the project CNP0000362. The Whole Genome Shotgun project has been deposited at NCBI under the accession number JADBJO000000000 (Biosample: SAMN16261191; Bioproject: PRJNA665547). The version described in this paper is JADBJO010000000. The transcriptome reads have been previously deposited in the NCBI SRA database with accession numbers SRS1009725 (for the Big dataset), SRS1009729 (for the Middle dataset), SRS1009726 (for the Small dataset), SRS1009727 (for the Normalized dataset), and SRS1009728 (for the Bulb dataset). The clean reads for 11,026 clones were submitted to the NCBI as EST data (ID: PRJNA290540). All the mass spectrometry proteomics data have been submitted to the ProteomeXchange Consortium (http://www.proteomexchange.org/) via the PRIDE partner repository with the dataset ID PXD014892.
