# Supplementary material for: The first Conus genome assembly reveals a primary genetic central dogma of conopeptides in C. betulinus
Source: Cell Discov. 2021 Feb 23;7:11. doi: 10.1038/s41421-021-00244-7 (PMC7900195; doi:10.1038/s41421-021-00244-7)
Supplement: Supplementary file 1 — Supplementary information [file 41421_2021_244_MOESM1_ESM.docx]

Supplementary Materials for

**The first *Conus* genome assembly reveals a primary genetic central dogma of conopeptides in *C. betulinus***

Chao Peng^1,2,†^, Yu Huang^1,3,†^, Chao Bian^1,3,4,†^, Jia Li^1,†^, Jie Liu^5,6,†^, Kai Zhang^1,3,10,†^, Xinxin You^1,3^, Zhilong Lin^5^, Yanbin He^5^, Jieming Chen^1,3^, Yunyun Lv^1,3,11^, Zhiqiang Ruan^1,3^, Xinhui Zhang^1^, Yunhai Yi^1,3^, Yanping Li^1,11^, Xueqiang Lin^1^, Ruobo Gu^1^, Junmin Xu^1^, Jiaan Yang^7^, Chongxu Fan^8^, Ge Yao^8^, Ji-Sheng Chen^8^, Hui Jiang^8^, Bingmiao Gao^2,^*, Qiong Shi^1,3,9,^*

^1^Shenzhen Key Lab of Marine Genomics, Guangdong Provincial Key Lab of Molecular Breeding in Marine Economic Animals, BGI Academy of Marine Sciences, BGI Marine, BGI, Shenzhen, Guangdong, China.

^2^Key Laboratory of Tropical Translational Medicine of Ministry of Education, Hainan Provincial Key Laboratory of Research and Development of Herbs, School of Pharmacy, Hainan Medical University, Haikou, Hainan, China.

^3^BGI Education Center, University of Chinese Academy of Sciences, Shenzhen, Guangdong, China.

^4^Center of Reproduction, Development and Aging, Faculty of Health Sciences, University of Macau, Macau, China.

^5^BGI-Shenzhen, BGI, Shenzhen, Guangdong, China.

^6^China National GeneBank, BGI, Shenzhen, Guangdong, China.

^7^Micro Pharmtech Ltd., Wuhan, Hubei, China.

^8^Research Institute of Pharmaceutical Chemistry, Beijing, China.

^9^Laboratory of Fish Genomics, College of Life Sciences and Oceanography, Shenzhen University, Shenzhen, Guangdong, China.

^10^Present address: College of Animal Science and Technology, Zhongkai University of Agriculture and Engineering, Guangzhou, Guangdong, China.

^11^Present address: College of Life Sciences, Neijiang Normal University, Neijiang, Sichuan, China.

† These authors contributed equally to this work.

* Correspondence: shiqiong@genomics.cn (Qiong Shi ), gaobingmiao@hainmc.edu.cn ( Bingmiao Gao )

**This file includes:**

Supplementary Tables S1 to S20

Supplementary Figs. S1 to S6

**Table S1.** Summary of the Illumina HiSeq sequencing data.

| **Insert size (bp)** | **Sequencing length (bp)** | **Raw data (Gb)** | **Clean data (Gb)** |
| --- | --- | --- | --- |
| 250 | 150 | 86.39 | 73.37 |
| 500 | 150 | 57.55 | 39.42 |
| 800 | 100 | 22.11 | 20.57 |
| 2,000 | 100 | 40.32 | 18.05 |
| 5,000 | 100 | 38.74 | 18.27 |
| 10,000 | 100 | 38.62 | 12.12 |
| 20,000 | 100 | 31.51 | 6.25 |
| Total | - | 315.24 | 188.05 |

**Table S2**. Summary of the PacBio sequencing data.

| **Library ID** | **Total bases (Gb)** | **Total reads** | **Average length (bp)** | **Max length (bp)** | **Min length (bp)** | **N50 (bp)** |
| --- | --- | --- | --- | --- | --- | --- |
| PacBio | 239.69 | 38,193,886 | 6,259.68 | 94,514 | 50 | 10,704 |

**Table S3**. Genome-size estimation based on the 17-mer frequencies

| **K-mer** | **K-mer number** | **K-mer depth** | **Genome size (bp)** | **Used base (bp)** | **Used read** | **×** |
| --- | --- | --- | --- | --- | --- | --- |
| 17 | 127,565,884,115 | 32 | 3,986,433,878 | 147,723,450,670 | 1,088,126,704 | 37.06 |

**Table S4**. Detailed classifications of the repeat sequences

| **Transposable element** | **Number** | **Base (bp)** | **% of the genome** |
| --- | --- | --- | --- |
| DNA transposon | | | |
| other | 569,715 | 79,019,237 | 2.30 |
| Sola | 1,168,979 | 252,996,348 | 7.37 |
| hAT | 1,725,743 | 227,644,024 | 6.64 |
| Novosib | 1,330,126 | 217,838,645 | 6.35 |
| CMC | 1,702,963 | 195,814,953 | 5.71 |
| Ginger | 782,469 | 108,853,600 | 3.17 |
| Kolobok | 486,582 | 93,668,487 | 2.73 |
| Helitron | 485,220 | 64,239,213 | 1.87 |
| Maverick | 325,423 | 37,753,258 | 1.10 |
| LTR Retrotransposon | | | |
| other | 110,072 | 15,414,367 | 0.45 |
| Gyps | 604,487 | 102,364,333 | 2.98 |
| ERV1 | 519,928 | 48,735,982 | 1.42 |
| ERVK | 325,213 | 27,834,920 | 0.81 |
| ERV | 134,737 | 21,551,494 | 0.63 |
| Copia | 174,048 | 21,120,999 | 0.62 |
| DIRS | 65,723 | 17,178,292 | 0.50 |
| LINE | | | |
| Other | 205,286 | 37,634,914 | 1.10 |
| RTE | 681,349 | 132,383,121 | 3.86 |
| L2 | 471,297 | 83,140,911 | 2.42 |
| I | 416,595 | 81,478,453 | 2.37 |
| L1 | 399,815 | 61,941,521 | 1.81 |
| Penelope | 382,800 | 60,981,342 | 1.78 |
| Jockey | 158,061 | 36,578,540 | 1.07 |
| R2 | 111,273 | 32,578,819 | 0.95 |
| CR1 | 132,925 | 27,794,227 | 0.81 |
| SINE | 42,476 | 14,136,091 | 0.41 |
| Satellite | 605,619 | 58,389,029 | 1.70 |
| Simple repeat | 1,354,087 | 209,548,443 | 6.11 |
| Unclassified | 2,403,437 | 471,192,390 | 13.73 |
| Total (Non-redundant) | 15,333,970 | 1,322,944,460 | 38.56 |

**Table S5**. Functional assignments of annotated genes from our genome assembly*.*

| **Parameter** | **Number** | **Percentage (%)** |
| --- | --- | --- |
| Total | 22,698 | 100.00 |
| Nr | 19,190 | 84.54 |
| Swissprot | 14,530 | 64.01 |
| KEGG | 15,464 | 68.13 |
| COG | 5,294 | 23.32 |
| TrEMBL | 19,188 | 84.54 |
| Interpro | 12,860 | 56.66 |
| GO | 10,251 | 45.16 |
| Annotated | 19,775 | 87.12 |

**Table S6.** Conopeptide genes *conot001-133*. Nucleotide sequence(s) of the exon(s) in each conopeptide gene are provided for public availability. The numbers between exon sequences are the lengths of introns. Potentially corresponding relationships between genes and transcripts are predicted.

(*See the separate file*)

**Table S7.** Deduced proteins CONOT001-133 (encoded by the genes *conot001-conot133*). These conopeptide proteins were categorized into various superfamilies and groups based on ConoServer (http://www.conoserver.org/) and our previous report (Peng C. et al. *GigaScience* **5**, 17 (2016)). Potentially corresponding relationships between proteins and transcripts are provided.

(*See the separate file*)

**Table S8**. Mapped conopeptide genes on the 35 groups of superscaffolds.

| Superscaffold | Conopeptide Gene | | | | |
| --- | --- | --- | --- | --- | --- |
|  | **position 1** | **position 2** | **position 3** | **position 4** | **position 5** |
| Group2 | conot031* | conot075 | conot080 |  |  |
| Group3 | conot007 | conot009 | conot058(conot056, conot057) | conot114 |  |
| Group4 | conot131 |  |  |  |  |
| Group6 | conot012 | conot046*(conot045*) | conot047* | conot049* | conot103 |
| Group11 | conot133 |  |  |  |  |
| Group12 | conot014 | conot016 | conot102 |  |  |
| Group13 | conot042 | conot067(conot066) |  |  |  |
| Group14 | conot018 | conot063(conot062) |  |  |  |
| Group15 | conot003 | conot004 |  |  |  |
| Group19 | conot072 | conot077 | conot078 |  |  |
| Group22 | conot071* | conot073*（conot074*） | conot079 |  |  |
| Group23 | conot036 |  |  |  |  |
| Group24 | conot076 |  |  |  |  |
| Group25 | conot030(conot026, conot027) | |  |  |  |
| Group27 | conot041 | conot064* | conot128* |  |  |
| Group35 | conot032 | conot033 | conot040 |  |  |

Note: Genes with * are short of 1-2 exons; Genes in brackets are comparable to those before the brackets at the same position, indicating that they are highly similar.

**Table S9.** cDNA sequences of 123 conopeptide transcripts identified from the 'Normalized' dataset of a middle body-sized specimen. They were summarized from our previous transcriptome report (Peng C. et al. *GigaScience* **5**, 17 (2016)).

(*See the separate file*)

**Table S10.** Conopeptide genes and transcripts expressed in individual middle body-sized *C. betulinus*. Blast parameters were restricted to 95% alignment length ratio and 95% identity.

(*See the separate file*)

**Table S11.** Numbers of conopeptide genes transcribed in the middle body-sized snail and mixed specimens per superfamily.

(*See the separate file*)

**Table S12.** Peptides detected by Q Exactive HF.

(*See the separate file*)

**Table S13.** Peptides detected by TripleTOF5600.

(*See the separate file*)

**Table S14.** FASTA sequences of 2,049 detected conopeptides (without consideration of any post-translational modifications). These peptides were detected by the two MS methods.

(*See the separate file*)

**Table S15.** A total of 2,474 detected venom conopeptides with post-translational modifications.

(*See the separate file*)

**Table S16.** Summary of the detected peptides and 142 recovered conopeptide proteins.

(*See the separate file*)

**Table S17**. The reported conopeptides with anti-addictive activities.

| **Conotoxin** | **Sequence** | **Species** | | **Reference** | | |
| --- | --- | --- | --- | --- | --- | --- |
| AuIB | GCCSYPPCFATNPDC(NH2) | | *Conus aulicus* | | [1] |  |
| Conantokin-G | GEγγLQγNQγLIRγKSN(NH2) | | *Conus geographus* | | [2] |  |
| Conantokin-G [S^16^Y] | GEγγLQγNQγLIRγKYN(NH2) | | *Conus geographus* | | [2] |  |
| Conantokin-G [γ^7^K] | GEγγLQKNQγLIRγKSN(NH2) | | *Conus geographus* | | [2] |  |
| Conantokin-G [γ^3^E,γ^4^E,γ^7^E,γ^10^E,γ^14^E] | GEEELQENQELIREKSN(NH2) | | *Conus geographus* | | [3,4] |  |
| Conantokin-G [1-11][γ^3^E,γ^4^E,γ^7^E,γ^10^E] | GEEELQENQEL | | *Conus geographus* | | [3,4] |  |
| CVID | CKSKGAKCSKLMYDCCSGSC SGTVGRC(NH2) | | *Conus catus* | | [5] |  |
| LsIA [R^10^F, N^12^L] | SGCCSNPACFVLNPNIC(NH2) | | *Conus limpusi* | | [6] |  |
| Lt14a | MCPPLCKPSCTNC(NH2) | | *Conus litteratus* | | [7] |  |
| MII [H^9^A, L^15^A] | GCCSNPVCALEHSNAC(NH2) | | *Conus magus* | | [8] |  |
| RegIIA [N^11^A, N^12^A] | GCCSHPACNVAAPHIC(NH2) | | *Conus regius* | | [9] |  |
| TxIB | GCCSDPPCRNKHPDLC(NH2) | | *Conus textile* | | [10] |  |

γ: Gamma carboxylic glutamic acid

[1] Muldoon, P.P. et al. *Br. J. Pharmacol.* 171, 3845-3857 (2014).

[2] Wei, J. et al. *Neurosci. Lett.* **405**, 137-141 (2006).

[3] Li, J. et al. *Chin. J. Drug Depend.* **22**(3), 182-187 (2013).

[4] Yuan, F. & Zhu, Y. *Chin. J. Drug Depend.* **22**(1), 4-10 (2013).

[5] Kolosov, A. et al. *Pain Med.* **12**, 923-941 (2011).

[6] Abraham, N. et al. *Sci. Rep.* **7**, 45466 (2017).

[7] Ren, Z. et al. *Toxicon* **96**, 57-67 (2015).

[8] Jackson, K.J.et al. *J. Pharmacol. Exp. Ther.* **331**, 547-554 (2009).

[9]Kompella, S.N. et al. *J. Biol. Chem.* **290**, 1039-1048 (2015).

[10]Zhangsun, D. et al. *Chin. Pharm. J.* **52**(7), 574-580 (2017).

**Table S18**. Protein sequence alignments of homologous conopeptides.

| **Name** | **Conopeptide Sequence** | **GenBank Accession No.** |
| --- | --- | --- |
| **(A) *Analgesic Activity*** | | |
| **MVIIA** ^a^ | -CKGKGAKCSRLMYDCCTGSCRS--GKC*-- | P05484.2 |
| **CONOT097** | -CLARGSRCN-YSSQCCSSFCRRRMGKCYY- | --- (Present study) |
| **Bt168** | -CLASGSWCE-YSTQCCSTYCRHRVHKCA-- | KU563979.1 |
| **Bt175** | -CLPSGSQCN-YSTQCCTTYCRR--HKCA-- | KU564050.1 |
| **Bt134** | -CLLNGEPCVPINGDCCSGVCVI---ICVP- | KU563960.1 |
| **CONOT089** | TCTPPGGLCG--YKNCC-GVCYAVINTCA-- | --- (Present study) |
| **Bt144** | TCTPPGGLCG-FYKNCC-GVCYAVINTCA-- | KU563969.1 |
| **(B) *Antiaddictive Activity*** | | |
| **AuIB** ^b^ | --GCCSYPPCFATNPD-C*- | P56640.2 |
| **Bt007** | -GGCCSYPPCIASNPK-CG- | KU564009.1 |
| **Bt004** | SATCCNYPPCYETYPESCL- | KU564008.1 |
| **Bt008** | NAECCYYPPCYEAYPEICL- | KU317629.1 |
| **Bt001** | -GGCCSHPACGVNHPELC-- | KU563886.1 |
| **Bt006** | -RGCCSHPACSVNHPELC-- | KU563888.1 |
| **Bt027** | -DDCCPDPACRQNHPELCSS | KU564013.1 |
| **(C) *Insecticidal Activity*** | | |
| **ImI** ^c^ | --GCCSDPRCAW-R---C*- | KJ801971.1 |
| **Bt001** | -GGCCSHPACGVNHPELC*- | KU563886.1 |
| **Bt006** | -RGCCSHPACSVNHPELC-- | KU563888.1 |
| **Bt027** | -DDCCPDPACRQNHPELCSS | KU564013.1 |
| **Bt002** | -GGCCSYPACSVEHQDLCD- | KU564007.1 |
| **Bt007** | -GGCCSYPPCIASNPK-CG- | KU564009.1 |
| **Bt004** | SATCCNYPPCYETYPESCL- | KU564008.1 |

Note: Conserved residues (with similar properties) among different conopeptides are highlighted in the same background color.

*, Amidated C-terminus.

^a^ A omega-conopeptide with the commercial name of Ziconotide. It is a selective blocker of neuronal N-type voltage-sensitive calcium channels (Wang Y.-X. et al. *Pain* **84**, 151-158 (2000)).

^b^ A alpha-conopeptide that selectively blocks α3β4 nicotinic acetylcholine receptors (Luo, S. et al. *J. Neurosci.* **18**, 8571-8579 (1998)).

^c^ A alpha-conopeptide that can block α7 nicotinic acetylcholine receptors (Yu, R. et al. *PLoS Comput. Biol.* **7**, e1002011 (2011)).

**Table S19**. Transcription levels of conopeptides Bt018 and Bt176 (Peng, C. et al. *GigaScience* **5**, 17 (2016)).

| **Conotoxin** | **Big** | |  | **Middle** | |  | **Small** | |
| --- | --- | --- | --- | --- | --- | --- | --- | --- |
|  | **RPKM** | **Ranking** |  | **RPKM** | **Ranking** |  | **RPKM** | **Ranking** |
| Bt018 | 57,233 | 3/95 |  | 71,975 | 2/94 |  | 32,503 | 2/98 |
| CONOT103(Bt176) | — | — |  | — | — |  | 2,087 | 27/98 |

RPKM: Reads Per Kilobase per Million mapped reads.

**Table S20**. Digestive enzymes and cleavage sites for the representative conopeptide CONOT019(Bt025).

| **Name of the digestive enzyme** | **Number of cleavages** | **Position of the cleavage site(s)** |
| --- | --- | --- |
| [Arg-C proteinase](https://web.expasy.org/peptide_cutter/peptidecutter_enzymes.html#ArgC) | 5 | 25, 41, 65, 70, 86 |
| [Asp-N endopeptidase](https://web.expasy.org/peptide_cutter/peptidecutter_enzymes.html#AspN) | 3 | 28, 59, 74 |
| [Asp-N endopeptidase + N-terminal Glu](https://web.expasy.org/peptide_cutter/peptidecutter_enzymes.html#AspGluN) | 4 | 28, 59, 74, 81 |
| [CNBr](https://web.expasy.org/peptide_cutter/peptidecutter_enzymes.html#CNBr) | 1 | 74 |
| [Chymotrypsin-high specificity (C-term to [FYW], not before P)](https://web.expasy.org/peptide_cutter/peptidecutter_enzymes.html#Ch_hi) | 3 | 32, 59, 79 |
| [Chymotrypsin-low specificity (C-term to [FYWML], not before P)](https://web.expasy.org/peptide_cutter/peptidecutter_enzymes.html#Ch_lo) | 8 | 32, 46, 53, 58, 59, 74, 78, 79 |
| [Clostripain](https://web.expasy.org/peptide_cutter/peptidecutter_enzymes.html#Clost) | 5 | 25, 41, 65, 70, 86 |
| [Formic acid](https://web.expasy.org/peptide_cutter/peptidecutter_enzymes.html#HCOOH) | 2 | 29, 75 |
| [Glutamyl endopeptidase](https://web.expasy.org/peptide_cutter/peptidecutter_enzymes.html#Glu) | 1 | 72 |
| [LysC](https://web.expasy.org/peptide_cutter/peptidecutter_enzymes.html#LysC) | 2 | 43, 73 |
| [LysN](https://web.expasy.org/peptide_cutter/peptidecutter_enzymes.html#LysN) | 2 | 42, 72 |
| [NTCB (2-nitro-5-thiocyanobenzoic acid)](https://web.expasy.org/peptide_cutter/peptidecutter_enzymes.html#NTCB) | 3 | 55, 76, 80 |
| [Pepsin (pH1.3)](https://web.expasy.org/peptide_cutter/peptidecutter_enzymes.html#Pn1.3) | 8 | 31, 52, 53, 57, 58, 59, 77, 78 |
| [Pepsin (pH>2)](https://web.expasy.org/peptide_cutter/peptidecutter_enzymes.html#Pn2) | 11 | 31, 52, 53, 57, 58, 59, 77, 78, 79, 83, 90 |
| [Proteinase K](https://web.expasy.org/peptide_cutter/peptidecutter_enzymes.html#ProtK) | 15 | 24, 27, 32, 52, 53, 54, 55, 58, 59, 72, 76, 78, 79, 80, 83 |
| [Staphylococcal peptidase I](https://web.expasy.org/peptide_cutter/peptidecutter_enzymes.html#Staph) | 1 | 72 |
| [Thermolysin](https://web.expasy.org/peptide_cutter/peptidecutter_enzymes.html#Therm) | 10 | 31, 51, 52, 53, 57, 58, 70, 73, 77, 87 |
| [Trypsin](https://web.expasy.org/peptide_cutter/peptidecutter_enzymes.html#Tryps) | 7 | 25, 41, 43, 65, 70, 73, 86 |


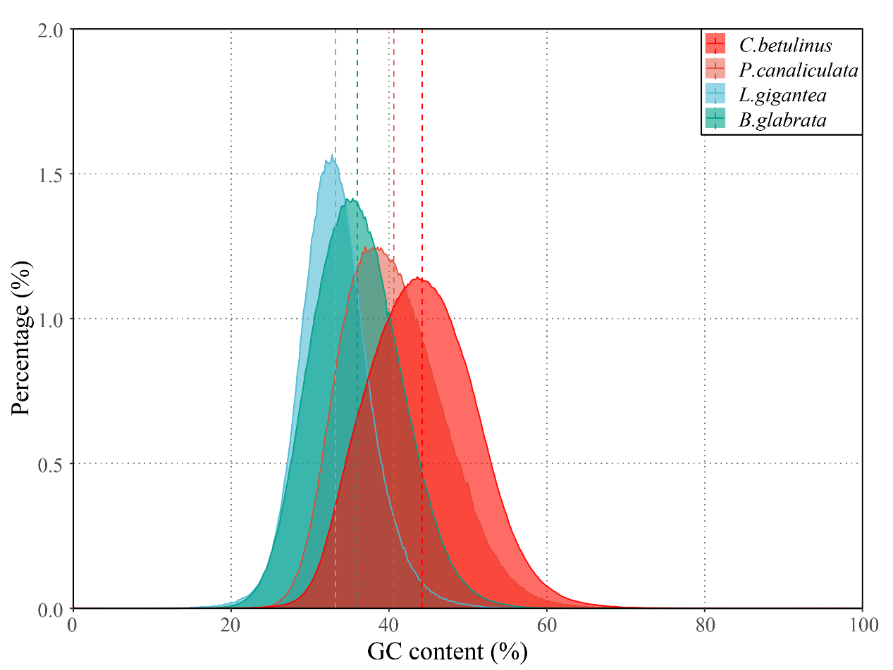


**Fig. S1 Comparisons of GC content between *C. betulinus* and other three published snails.** Values in this plot were calculated by sliding 500-bp non-overlapping windows against corresponding genome sequences. The x-axis represents the GC content (%), and the y-axis represents the percentage of each GC content. The examined other three snail species include Apple snail (*Pomacea canaliculata*), Giant owl limpet (*Lottia gigantea*), and Bloodfluke planorb (*Biomphalaria glabrata*).


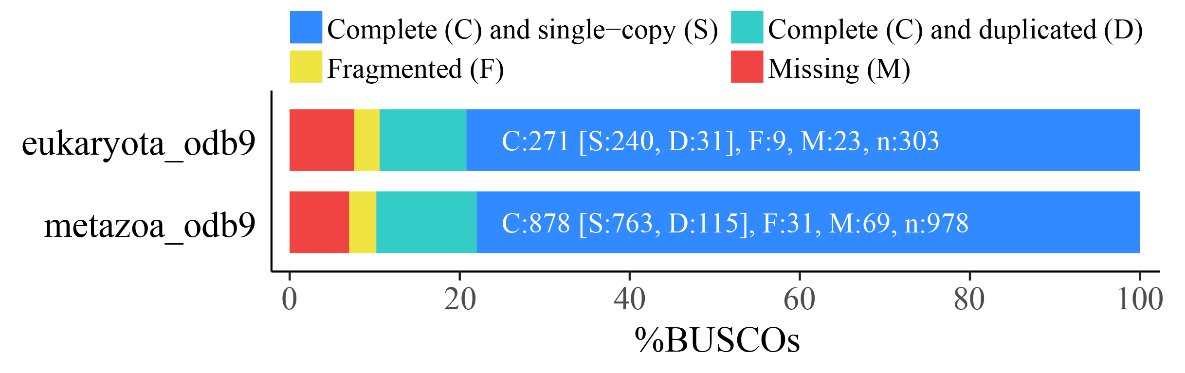


**Fig. S2 |** **A BUSCO assessment of our assembled genome of *C. betulinus***. We used two single-copy ortholog datasets, eukaryota_odb9 (303 BUSCO groups) and metazoan_odb9 (978 BUSCO groups), to evaluate the completeness and accuracy of our genome assembly. Our results support high-quality of the assembled genome.


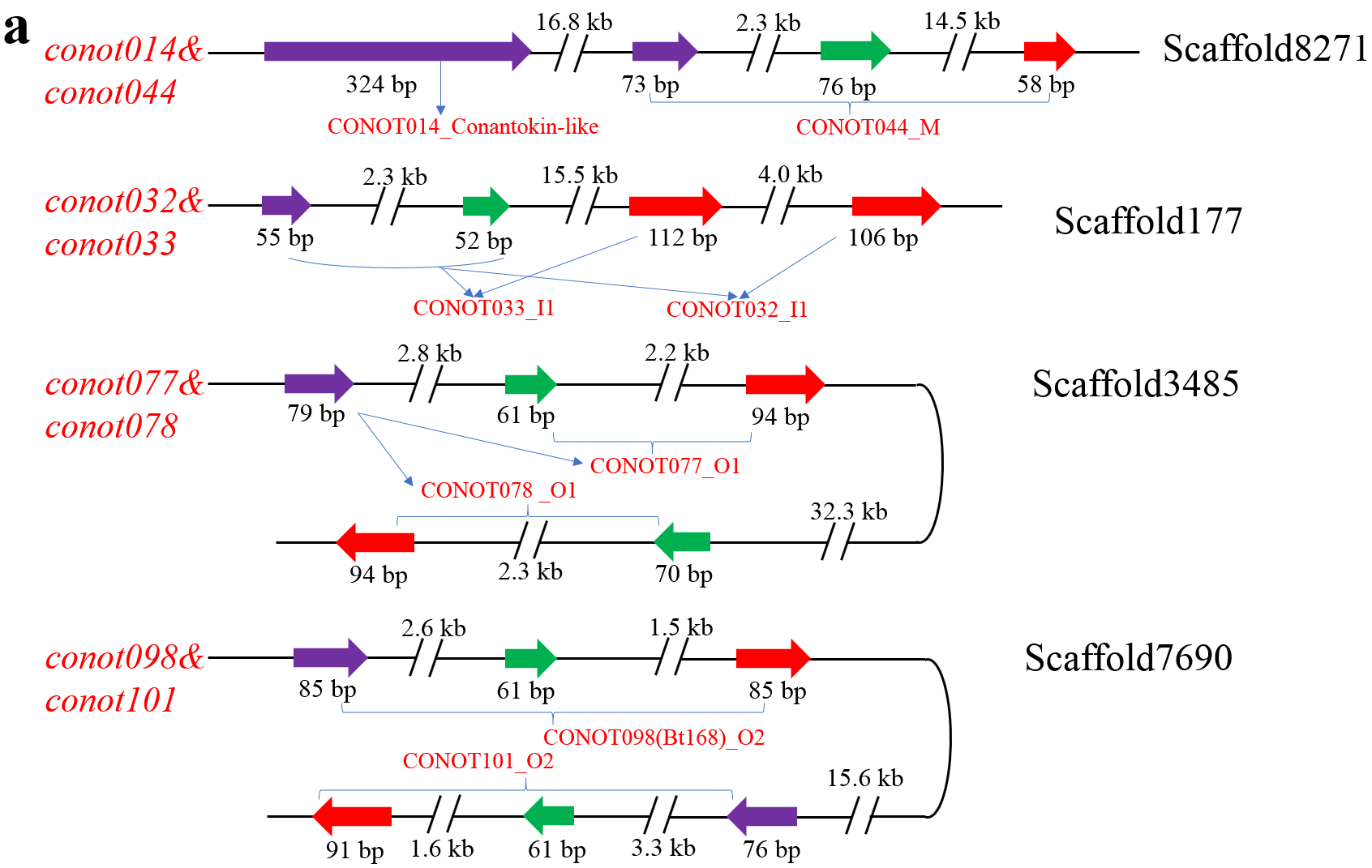

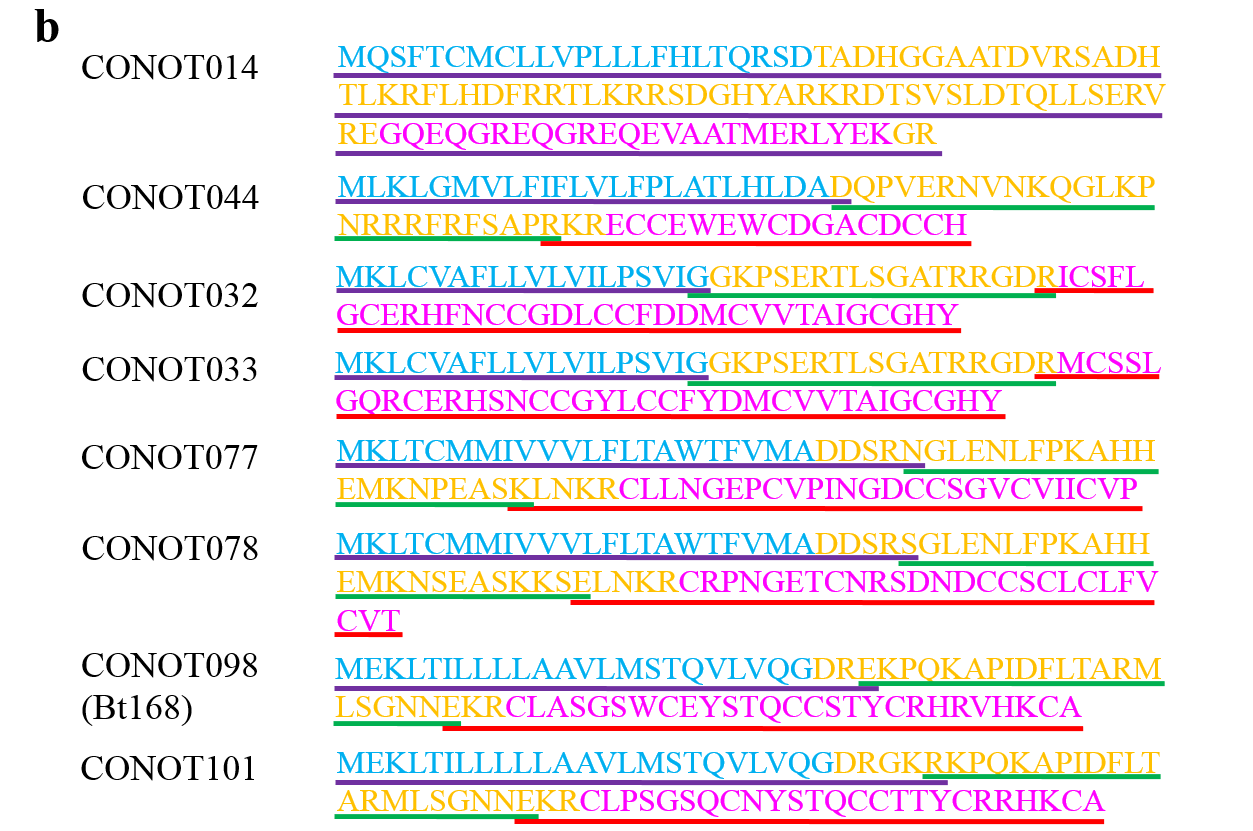


**Fig. S3 Exon distribution and protein sequences of representative conopeptide genes. a** Four representative types of exon distribution in the identified full-length conopeptide genes. Regions in purple, green, red and black colors represent the first, second, third and fourth exons, respectively. **b** Protein sequences of the intact genes in (**a**). Sequences highlighted in black, yellow, and red are signal peptides, pro-/post-peptides, and mature peptides, respectively.


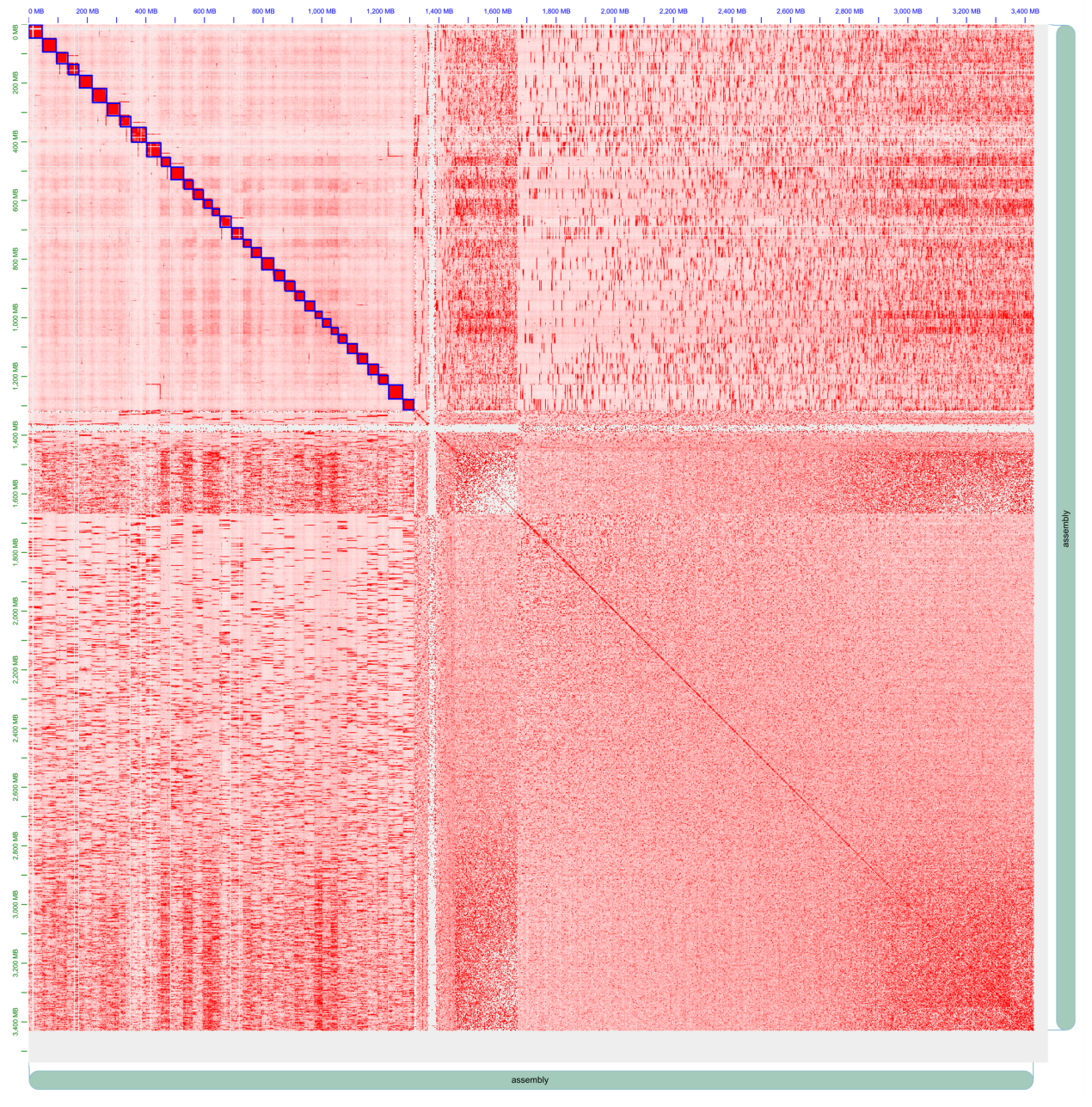


**Fig. S4 A Hi-C global heatmap for *C. betulinus* reference genome showing interactions between the 35 groups of superscaffolds.** The global heatmap (resolution 1.25 Mb) was taken from the screenshot in JuiceBox. Each blue box represents one of the 35 large groups. The bases in contigs are 38.60% of the achieved genome assembly.


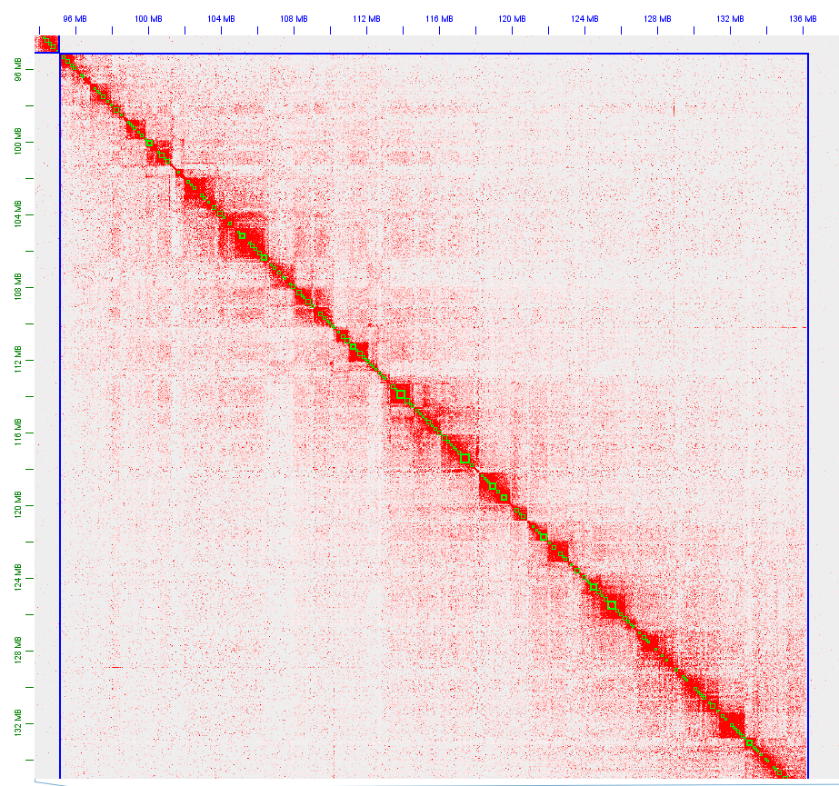


**Fig. S5 A Hi-C interaction heat map for *C. betulinus* reference genome showing interactions between the 35 groups of superscaffolds.** The interaction heatmap (resolution 25 kb) with a range between 94 Mb to 136 Mb for each chromosome was taken from the screenshot in JuiceBox. The large blue box on the outside represents a group and each small green box inside represents one contig. The denser red dots, the stronger interaction in the area.


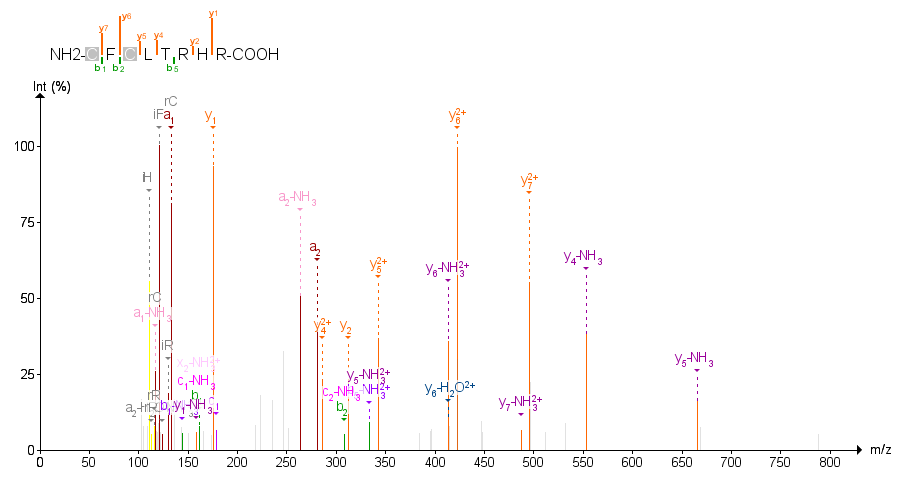


**Fig. S6 A detailed PSM map for BT112.** Six y-ions (orange peaks) and three b-ions (green peaks) were identified for the short peptide BT112 (CFCLTR) from the MS/MS spectra. The PSM image was visualized by PDV, an integrative proteomics data viewer (Li C. et al. *Bioinformatics* **35**, 1249-1251 (2019)).
